# Supplementary material for: Phenotype-Based and Self-Learning Inter-Individual Sleep Apnea Screening With a Level IV-Like Monitoring System
Source: Front Physiol. 2018 Jul 2;9:723. doi: 10.3389/fphys.2018.00723 (PMC6036126; doi:10.3389/fphys.2018.00723)
Supplement: Supplementary file 1 [file Presentation_1.pdf]

SUPPLEMENTARY MATERIAL

1. METRIC DESIGN

In this section, we detail the designed phenotype-base metric to determine the similarity between subjects, and hence the *modified KNN scheme*. We consider the commonly available phenotype information for each subject, including gender, age, and body-mass index (BMI). It is well-known that gender, age and BMI are closely related to the sleep apnea pattern and severity [1, 2]. The gender is saved as 0 and 1 to indicate male and female. While there is no ground truth guiding this similarity, we take the physician's clinical experience to design the metric. We weight the standardized Euclidean distances between the gender, BMI and age by 4, 2 and 1 to form the phenotype distance between subjects; that is, we view gender as the most important parameter, followed by BMI and then age. In clinics, the gender, BMI and age are not the only considered parameters related to the SAS severity. The comorbidity of hypertension, diabetes, and hypothyroidism are all relevant. To further take physicians' wisdom into account, we take the comorbidity of hypertension [3], diabetes [4], and hypothyroidism [5] into account to better determine the similarity between subjects. We view comorbidity as a categorical variable, and save it as 1 and 0 to indicate the presence of a disease. The Euclidean distance between the vectors saving co-morbidity status of two subjects is called the correction distance. Following the clinical practice, we can find the K nearest neighbors (KNN). If we want to find the K most similar subjects of the new-arriving subject, we first determine $K+K'$ most similar subjects that are related to the phenotype metric, and remove the K' subjects with the largest correction distance. If there are less than K' subjects that have the correction distance greater than 0, we remove subjects with the largest phenotype distance to determine the K most similar subjects. This is the *modified KNN scheme* considered in the main article.

2. SELECTED FEATURES

In this work, we consider features extracted from the thoracic movement recorded from the triaxial accelerator sensor (TAA-THO), the abdominal movement recorded from the triaxial accelerator

sensor (TAA-ABD) [6], and the peripheral capillary oxygen saturation (SpO_2). We extract apnea-related features from TAA-THO and TAA-ABD, and the desaturation features from SpO_2. For the calibration between PSG and sensors, the calibration procedure is done by taking the PSG signals during initial tests, like normal-stop breaths and left-right turns, as the reference. The calibrated TAA signals are then treated as the baseline for the following signal recording. However, the automatic calibration procedure has not been implemented.

2.1. **Features from the Respiratory Signal – apnea or not.** The proposed algorithm extracts the features from TAA-THO, denoted as$Y_{\texttt{tho}}$, and TAA-ABD, denoted as $Y_{\texttt{abd}}$, to identify apnea events. Based on the physiological phenomenon, two features, amplitude ratio (AR) and frequency ratio (FR) of TAA-THO and TAA-ABD, are used to identify apnea events [7].

TAA-ABD and TAA-THO are down-sampled from 226Hz to 4Hz. We segment the signals into overlapping windows of 10-s duration with 9.5-s overlap. We call these windows *current windows* (CW), and denote the n-th CW as $\texttt{CW}(n)$. The CW provide information about the apnea event, and the goal is to determine if there is a sleep apnea event over each CW. Due to the nature of the triaxial accelerator sensor and the possible body movement, when we evaluate the amplitude of TAA-THO and TAA-ABD, the amplitude might not be meaningful. Instead, it is found in [7] that the ratio of the amplitude in the current window and the previous window is more informative. Therefore, we consider another set of windows to define the feature. For each CW, we call the closest window of 60-s duration in which *no apnea* was reported by the sleep expert the *pre-window* (PW). The TAA-THO and TAA-ABD over PW contains the *baseline* information for the amplitude information. Denote $\texttt{PW}(n)$ to be the n-th PW associated with the n-th CW. Note that PW's might be the same for different CW's, particularly during the apnea event. In other words, the PW is fixed when the CW moves forward, if there is a sleep apnea on the CW annotated by the sleep expert. The relationship between CW and PW is illustrated in Figure 1.

In this study, the lengths of PW and CW, 60 and 10 seconds separately, are chosen based on the rules used by the sleep expert to mark apnea events. In practice, the sleep expert compares the amplitudes of the oral-nasal flow and the abdominal and thoracic movement signals in the current 10 s with those of the previous signals, up to 120 s, to determine an event. To reduce the possibility of information mixup, we selected 60-s duration as our PW.

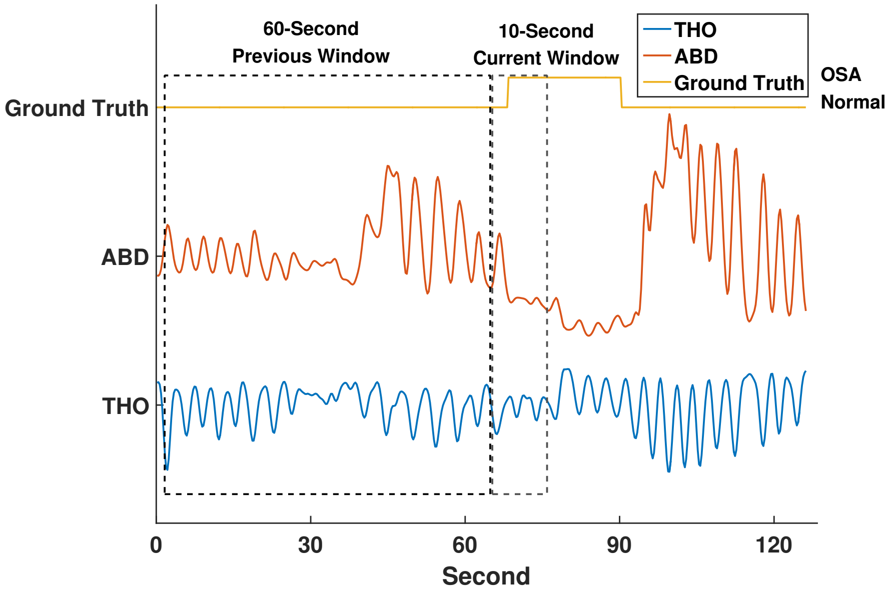

FIGURE 1. An illustration of the 60-second previous window and 10-second current window used in the feature extraction scheme.

We followed the suggested nonlinear filtering technique, the synchrosqueezing transform [7, Equation (2)], to quantify the amplitude of the recorded TAA-THO and TAA-ABD signals, denoted as $A_{\texttt{tho}}$ and $A_{\texttt{abd}}$. AR features capture the *attenuation* of the respiratory signal. The AR feature of the TAA-THO over the n-th CW is defined as the ratio of the 95% quantile of the amplitude over $\texttt{CW}(n)$ and that over $\texttt{PW}(n)$. Similarly, we could define the AR feature of the TAA-ABD over the n-th CW. The 95% quantile, instead of the maximum amplitude, is chosen to avoid outliers in the signal.

FR features quantity the *oscillatory pattern* of the respiratory signal. When the subject has a normal breathing pattern, the spectrum should be well concentrated in the low frequency region. When apnea events happen, the distorted respiratory pattern in the time domain causes a different concentration in the spectrum. The FR of TAA-THO over the n-th CW is defined as the log of the ratio of the TAA-THO energy over the frequency band ranging from 0.8Hz to 1.5Hz and that over the frequency band ranging from 0.1Hz to 0.8Hz. The FR of TAA-ABD over the n-th CW is defined in the same way. We follow the suggestion in [7] and take the integration range from 0.8 to 1.5 Hz, which might catch the cardiogenic artifact in the TAA signal, if there is any. Note that the PW is not used when we define FR.

2.2. **Feature between thoracic and abdominal movement – paradoxical movement.** In addition to the respiratory activity index, we quantified the paradoxical movement by using the covariance between TAA-THO and TAA-ABD signals over `CW`. The feature is called `Cov`. The paradoxical movement is a significant respiratory characteristic of the OSA event. However, it is not considered as the main feature used to distinguish apnea events since it does not occur in all OSA events. Like what sleep experts do, we use `Cov` as an auxiliary feature to further confirm whether a given apnea event is obstructive.

2.3. **Features from SpO_2 oxygen – desaturation or not.** Six features are extracted from SpO_2 for the apnea detection. These features are obtained by evaluating the minimum, maximum, median, mean, variance of the first derivative, and difference between median and minimum over a sliding 20-second window.

Note that physiologically there is a latency between an apnea/hypopnea event and its associated oxygen desaturation event. Therefore, we need to shift all features our oxygen desaturation detection result. Compare the sensitivity of detecting the oxygen desaturation events over the whole group, the optimal latency time is 20 seconds, when the mean of the sensitivities of all subjects is maximal, and the variance is minimal.

3. Learning Apnea Classifiers by the Support Vector Machine

For the new-arriving subject x, we find K subjects from $\mathscr{X}$ that are most similar to x in the sense of D. To learn the relationship between the apnea events and the extracted respiratory features from those K subjects for the prediction purpose, the kernel support vector machine (SVM) [8, 9] is considered based on the standard radial based function.

The respiration status determined by sleep experts based on the 2007 guidelines of American Academy of Sleep Medicine (AASM) is referred to as the ground truth in the training process, which we call the *PSG-state*. The PSG-states are evaluated every 0.5 second, and there are three label, normal NOR, apnea APN and hypopnea HYP. Here we do not distinguish between the central sleep apnea and obstructive sleep apnea . Therefore, we obtain a time series with the range $\{\mathsf{NOR}, \mathsf{APN}, \mathsf{HYP}\}$, which is denoted as $s_{\mathtt{PSG}}$.

Denote a ten dimensional vector $v(n)$ to be the features extracted from TAA-THO, TAA-ABD, and SpO_2 on $\texttt{CW}(n)$, which include $\texttt{AR}_{\texttt{tho}}$, $\texttt{FR}_{\texttt{tho}}$, $\texttt{AR}_{\texttt{abd}}$, $\texttt{FR}_{\texttt{abd}}$, and six desaturation features. Call $v(n)$ the *SAS feature* over $\texttt{CW}(n)$. The SAS features are divided into three groups, denoted as $\mathscr{C}$, $\mathscr{A}$ and $\mathscr{H}$, by the following rules:

(1) If $s_{\texttt{PSG}}$ is dominated by NOR over $\texttt{CW}(n)$, $v(n) \in \mathscr{N}$;

(2) If $s_{\texttt{PSG}}$ is dominated by APN over $\texttt{CW}(n)$, $v(n) \in \mathscr{A}$;

(3) If $s_{\texttt{PSG}}$ is dominated by HYP over $\texttt{CW}(n)$, $v(n) \in \mathscr{H}$.

Clearly, $\mathscr{N}$, $\mathscr{A}$, and $\mathscr{H}$ are associated with three different respiratory patterns. Three binary SVM classifiers are trained on $\mathscr{N}$, $\mathscr{A}$, and $\mathscr{H}$: the classifier **CLF_NA** is trained to classify $\mathscr{N}$ and $\mathscr{A}$, the classifier **CLF_NH** is trained to classify $\mathscr{N}$ and $\mathscr{H}$, and the classifier **CLF_AH** is trained to classify $\mathscr{A}$ and $\mathscr{H}$.

4. PREDICT APNEA EVENTS BY THE STATE MACHINE

We apply the SVM classifiers and $\texttt{Cov}$ to design a state machine for an online prediction system. Like the designed features, the state machine is designed to be as simple as possible to demonstrate the notion. There are three status, NOR, APN and HYP and four rules guiding the state transition, (1)-(4), stated below. The state machine initially stays in the NOR state, and outputs a prediction of sleep apnea events every 0.5 second.

The rules depend on the established binary SVM classifiers, **CLF_NA**, **CLF_NH**, and **CLF_AH** and the status of the previous L $\texttt{CW}$'s, where $L \in \mathbb{N}$. We call L the *tap number* for the state machine. While the tap number is a tunable parameter, following the suggestion in [7, Section V.B], the tap number is fixed to $L = 12$ in this study. At the n-th $\texttt{CW}$, depending on corresponding status, the transition occurs according to the following four rules, (1)-(4).

(1) if one of these conditions hold, transition from NOR to APN happened.
   (a) More than half **CLF_NH**(i), $n-L+1 \leq i \leq n$, are HYP, and **CLF_NA**$(i) = \mathsf{AH}$ and **CLF_AH**$(i) = \mathsf{A}$ for all $n-L+1 \leq i \leq n$.
   (b) More than half **CLF_NA**(i), $n-L+1 \leq i \leq n$, are AH, and **CLF_NH**$(i) = \mathsf{HYP}$, and **CLF_AH**$(i) = \mathsf{A}$ for all $n-L+1 \leq i \leq n$.

(2) **CLF_NA**$(i) = \mathsf{NOR}$ for all $n-L+1 \leq i \leq n$, transition from APN to NOR happened.

(3) if one of these conditions hold, transition from NOR to HYP happened.

(a) More than half $\mathbf{CLF\_NH}(i)$, $n-L+1 \leq i \leq n$, are HYP, and $\mathbf{CLF\_NA}(i) = \mathsf{AH}$ and $\mathbf{CLF\_AH}(i) = \mathsf{HYP}$ for all $n-L+1 \leq i \leq n$.

(b) More than half $\mathbf{CLF\_NA}(i)$, $n-L+1 \leq i \leq n$, are AH, and $\mathbf{CLF\_NH}(i) = \mathsf{HYP}$ and $\mathbf{CLF\_AH}(i) = \mathsf{HYP}$ for all $n-L+1 \leq i \leq n$.

(4) $\mathbf{CLF\_NH}(i) = \mathsf{NOR}$ for all $n-L+1 \leq i \leq n$, transition from HYP to NOR happend.

References

[1] O. Ogretmenoglu, A. Suslu, O. Yucel, T. Onerci, and A. Sahin, "Body fat composition: a predictive factor for obstructive sleep apnea," *Laryngoscope*, vol. 115, no. 8, pp. 1493–8, 2005.

[2] W.-T. Liu, H.-T. Wu, J.-N. Juang, A. Wisniewski, H.-C. Lee, D. Wu, and Y.-L. Lo, "Prediction of the severity of obstructive sleep apnea by anthropometric features via support vector machine," *PLOS One*, vol. 12, no. 5, p. e0176991, 2017.

[3] F. a. Javaheri, S. andBarbe, "Sleep apnea: Types, mechanisms, and clinical cardiovascular consequences," *J Am Coll Cardiol*, vol. 69, no. 7, pp. 841–858, 2017.

[4] B. Reutrakul, S. andMokhlesi, "Obstructivesleep apneaanddiabetes: A state of the artreview," *Chest*, vol. 152, no. 5, pp. 1070–86, 2017.

[5] P. J. Attal, P. andChanson, "Endocrine aspects of obstructivesleep apnea," *Clin Endocrinol Metab*, vol. 95, no. 2, pp. 483–95, 2010.

[6] J.-C. Wu, C.-W. Wang, Y.-H. Huang, H.-T. W. Wu, P.-C. Huang, and Y.-L. Lo, "A Portable Monitoring System with Automatic Event Detection for Sleep Apnea Level-IV Evaluation," in *2018 IEEE International Symposium on Circuits and Systems*, 2018.

[7] Y.-Y. Lin, H.-T. Wu, C.-A. Hsu, P.-C. Huang, Y.-H. Huang, and Y.-L. Lo, "Sleep apnea detection based on thoracic and abdominal movement signals of wearable piezo-electric bands," *Journal of Biomedical and Health Informatics, IEEE Journal of*, 2017, in press.

[8] A. Khandoker, M. Palaniswami, and C. Karmakar, "Support vector machines for automated recognition of obstructive sleep apnea syndrome from ecg recordings," *IEEE Trans. Inf. Technol. B.*, vol. 13, no. 1, pp. 37–48, 2009.

[9] H. Al-Angari and A. Sahakian, "Automated recognition of obstructive sleep apnea syndrome using support vector machine classifier," *IEEE Trans. Inf. Technol. B.*, vol. 16, no. 3, pp. 463–468, May 2012.
